# Collaborative Dishonesty: Children Are More Likely to Cheat When They Benefit Together

**DOI:** 10.1111/desc.70080

**Published:** 2025-10-13

**Authors:** Akzira Abuova, Laura Tietz, Sebastian Grueneisen

**Affiliations:** ^1^ Educational Faculty University of Leipzig Leipzig Germany

**Keywords:** cheating, collaboration, cooperation, culture, development, dishonesty

## Abstract

Collaboration, the process by which individuals work together toward mutual benefits, is a core feature of human sociality. Capacities for collaboration emerge early in development and represent an important social competence. Yet, collaborative commitments can conflict with commitments to societal norms such as honesty and rule compliance, and little is known about whether the development of collaborative proclivities can promote dishonesty in young children. In this study, we tested pairs of 6‐ to 8‐year‐old Kazakh‐speaking and Russian‐speaking children in Kazakhstan (*N* = 192), a Central Asian post‐Soviet society characterized by relatively high social interdependence compared to European and North American, post‐industrialized samples commonly recruited in developmental research. Children participated in a die‐rolling game in which they could misreport the frequency of a specific outcome to win rewards. We found that children were more likely to cheat when they benefited together with a partner (collaboration condition) than when they benefited individually (solo condition). Additionally, across both conditions, children in Kazakh‐speaking classes overreported winning outcomes more frequently than children in Russian‐speaking classes. These findings demonstrate that collaborative motives and socio‐cultural contexts can influence honesty from an early stage in development.

**Summary:**

- We investigated children's tendency to cheat in a game in which they could benefit together with a partner (collaboration condition) or benefit alone (solo condition).
- We tested 6‐ to 8‐year‐old Kazakh‐speaking and Russian‐speaking children from Kazakhstan.
- Children were more likely to cheat when they benefited together, suggesting that collaborative goals can compromise honesty early in development.
- Russian‐speaking children cheated less than Kazakh‐speaking children, highlighting the role of sociocultural factors in how children resolve norm conflicts.

## 1 Introduction

Humans are exceptionally cooperative (Bowles and Gintis 2011; Melis and Semmann 2010). Yet, across human societies dishonesty and corruption are widespread at substantial social, economic, and political costs (Dreher and Schneider 2010; Schneider and Buehn 2007; Tavits 2010). For example, corruption costs the world economy $2 trillion annually (Schwab 2013), leads to an erosion of trust in institutions (Enste and Heldman 2017; Rothstein and Uslaner 2005), causes environmental damage (Gardiner 2006; Welsch 2004), and affects subjective well‐being and health (Achim et al. 2020; Tay et al. 2014; Wu and Zhu 2016).

Neoclassical economics suggests dishonesty occurs when gains outweigh the risks of getting caught (Becker 1968; Leib et al. 2021), implying that self‐interest can override cooperative motives. However, many corrupt practices, such as bribery or price fixing, are inherently cooperative. An alternative view posits that people might sometimes act dishonestly not despite but because of their cooperative inclinations, for instance, when overlooking wrongdoing by friends or ingroup members (Weidman et al. 2020), when returning favors instead of being impartial (Niemi and Young 2017), or when cheating to support others (Shalvi and De Dreu 2014).

Indeed, adults consistently cheat more when their dishonesty promotes collaborative goals rather than personal gains (Leib et al. 2021; Pulfrey et al. 2018; Weisel and Shalvi 2015; Wiltermuth 2011). A common interpretation is that people treat collaboration as a moral currency that can be exchanged for honesty, offering individuals a justification for their transgressions while reducing the psychological costs of cheating (Weisel and Shalvi 2022). Moreover, cooperative interactions elicit a sense of obligation among collaborative partners (Michael et al. 2016; Tomasello 2020), and social closeness can shape moral wrongness judgments among US participants, thus potentially rendering dishonest acts supporting cooperative goals as subjectively permissible (Earp et al. 2021; McManus et al. 2020; Zickfeld et al. 2024).

While little is known about the developmental origins of collaborative dishonesty, children are motivationally and cognitively equipped to collaborate with others from an early age. Research with primarily North American and European children has shown that, by age 2‐3, children form joint goals, coordinate actions, and show an understanding of complementary roles in collaboration (Brownell et al. 2006; Warneken et al. 2006). By age 3‐4, they understand and act upon the commitments emanating from collaboration (Gräfenhain et al. 2009), for example, by expecting collaborative partners to remain engaged when tempted by appealing alternatives (Kachel et al. 2018) or by making sure collaborative partners benefit equally (Hamann et al. 2011). Moreover, 6‐year‐olds show a greater willingness to invest effort when they benefit together with a partner compared to when they benefit alone, further documenting the motivational force of mutual dependencies (Butler and Walton 2013; Koomen et al. 2020).

These early‐emerging collaborative skills are thought to lay the foundation for humans’ unique ability to flexibly achieve goals collectively that individuals could not accomplish alone (Tomasello et al. 2012). However, it remains unknown whether the development of collaborative tendencies also encourages dishonest behavior in young children.

Children first act dishonestly for personal gain around age 3, and their deception becomes increasingly sophisticated over the preschool and early school years (Evans and Lee 2013; Talwar and Lee 2002, 2008). By age 5, children show substantial flexibility in their application of truthfulness and deceit. For instance, Chinese children show more dishonesty when they believe it to be common or more acceptable (Liu et al. 2022) or when there are fewer physical obstacles (Zhao et al. 2022), but they cheat less when they promise honesty (Heyman et al. 2015). Around age 5, Chinese and North American children also begin behaving dishonestly to benefit others, for instance, by cheating in games (Zhao et al. 2019) or by telling lies to protect others’ feelings (Nagar et al. 2020; Warneken and Orlins 2015). Turkish children were more likely to tell prosocial lies in favor of individuals who had previously helped them or with whom they had completed a joint task (Aydin 2023). Moreover, 6‐year‐olds evaluate lies told to avoid personal trouble more negatively than lies meant to spare others’ feelings (Heyman et al. 2009) and trust prosocial liars just as much as truth‐tellers (Fu et al. 2015). However, prior research has not examined whether collaborative contexts in particular—where individuals stand to benefit together and commitments to one's partners can conflict with norms of honesty (Zickfeld et al. 2024)—encourage children to cheat. Addressing this question is the main goal of the current study.

To this end, we conducted an experiment with Kazakhstani children. Central Asian post‐Soviet countries remain among the most underrepresented regions in psychology (Krys et al. 2024), with the great majority of developmental research relying on North American and European samples (Nielsen et al. 2017). Similarly, prior work on collaborative dishonesty has mostly focused on adult participants from societies characterized by high levels of social independence and low levels of everyday rule violations (Leib et al. 2021).

Kazakhstan is a culturally, linguistically, and ethnically diverse country with a high degree of ethnic segregation (Popova and De Bot 2020; Rees and Williams 2017). Historically, Kazakhs, Kazakhstan's largest ethnic group, descend from nomadic groups whose traditional lifestyles and family structures place great emphasis on kinship, unity, and mutual support (Aimar 2019; Esenova 2002; Majidi et al. 2015), and a collectivist value orientation remains common (Adilova et al. 2021). Yet, many cultural influences pervade contemporary Kazakhstani society, with Russians—Kazakhstan's second largest ethnic group—and other Russian‐speakers (e.g., Ukrainians, Belarusians, and Germans) showing relatively more individualistic orientations (Zharkynbekova et al. 2025). While previous research with adults indicates that, despite a near universal emphasis on honesty across human societies, dishonesty for personal gain is negatively correlated with country‐level indices of institutional quality, corruption, and individualism (Cohn et al. 2019; Gächter and Schulz 2016; Payan et al. 2010), it is currently unclear whether this relationship extends to collaborative dishonesty or cheating rates of children.

Here, we tested Kazakh‐ and Russian‐speaking children who attended the same schools but were taught in different languages. Children thus experienced identical school curricula, came from the same urban areas, and shared most of their social‐demographic environment. We tested 6‐ to 8‐year‐olds since deceptive and collaborative abilities reach considerable sophistication at this age (Fawcett and Garton 2005; Grueneisen et al. 2015; Talwar and Lee 2008). Our preregistered main hypothesis was that children across language groups would be more likely to cheat in a collaborative context in which partners benefit together compared to a solo context in which they benefit alone. We also investigated whether cheating tendencies differed between children from Kazakh‐speaking and Russian‐speaking classes in the collaborative and the solo context. The current study thus aimed to elucidate the development of collaboratively motivated rule violations in an underrepresented population and to offer potential insights into the role of social‐cultural factors in shaping its emergence.

## 2 Methods

### 2.1 Participants

The final sample consisted of 192 children aged 6 to 8 (*M* = 7.08, SD = 0.76; 91 females, 93 from Kazakh‐speaking classes). Four additional children were tested but excluded because they failed to answer the comprehension check (*n* = 2) or due to a procedure error (*n* = 2). All participants were from a middle‐sized city (242,462 inhabitants) in south‐central Kazakhstan and were recruited from two local schools. Both schools were mixed language schools with separate Russian and Kazakh classes, which followed the same school curriculum. Participants came from diverse ethnic backgrounds. In the Kazakh‐speaking group, 98.9% of the children were ethnic Kazakhs and 1.08% Russians. 48.39% of parents reported that their child also used a language other than Kazakh in their daily life. In the Russian‐speaking group, 64.6% were Kazakhs, 14.1% Koreans, 11.1% Russians, 6.06% Uzbeks, and 4% others. 64.65% of parents reported that their child also used a language other than Russian in their daily life. For 11.98% of the children, data on daily language use was unavailable. The study was approved by the local Ministry of Education and the Ethics Advisory Board of Leipzig University. All parents gave written informed consent for their children to participate, and children gave verbal assent.

### 2.2 Setup and Procedure

Children were tested in a quiet room in their schools. Participants were randomly paired (gender‐matched) and assigned to the collaboration condition or the solo condition. Participants were tested in the language they were studying by a bilingual experimenter fluent in both languages. Scripts were drafted in English and translated into both languages by the first author and then re‐translated to English by native speakers unfamiliar with the research goals.

We used a modified version of the die‐rolling game developed by Fischbacher and Föllmi‐Heusi (2013)—one of the most widely used paradigms to study the motives underlying dishonesty (Abeler et al. 2019; Shalvi et al. 2025). A key strength of the paradigm is that it does not involve deception and instead allows researchers to infer cheating based on known probability distributions. In each of five rounds, children went behind a barrier and rolled five dice (that is, children rolled 25 dice in total). Each die had a star on one side and a dot on all others. Two stickers were awarded for each star that children reported rolling. The stickers were distributed in accordance with the respective condition. In the solo condition, children were independent and won stickers for themselves: for each star children reported to have rolled they received two stickers, and their partner was unaffected (and vice versa). In the collaboration condition, children benefited together: for each star children reported to have rolled, they and their partner received one sticker each. Since children were unobserved when playing the game, neither the experimenter nor the partner knew the true outcome of the die rolls. Children could thus cheat by overreporting the number of stars.

In both conditions, children were introduced to the game rules together, with the experimenter demonstrating how to roll the dice. Children then each played a practice round alone and with the experimenter watching. They were asked how many stickers they (solo condition) or they and their partner (collaboration condition) would win given the outcome they obtained, with the experimenter providing corrective feedback in case of a mistake. Before the first round, children in the collaboration condition were additionally asked: “How many stickers do you get if you roll three stars? And how many stickers does [partner name] get? How many stickers do you get if you roll only dots? And how many stickers does [partner name] get?” In the solo condition, children were asked: “How many stickers do you get if you roll three stars? And how many stickers do you get if you roll only dots?” Children who failed to answer these latter control questions were excluded.

**FIGURE 1:**
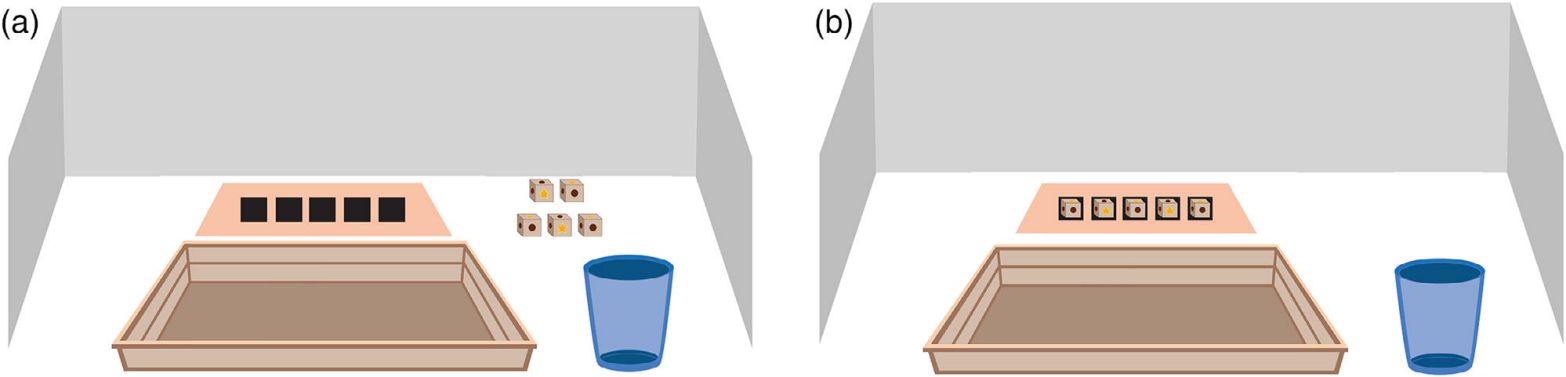
Game setup. (A) The setup before the child rolled the dice before each round. (B) Dice placed on designated spots on a sheet of paper by children after rolling all five dice. Children were asked to count the number of stars they rolled in total, put all dice back in the cup, and report the number of stars to the experimenter.

During the test rounds, children were invited to the room separately while their partner received a drawing task outside. The first child of the dyad went behind a barrier where the experimenter could not see them. Behind the barrier, they were asked to put the first die into a cup, roll the die on a wooden tray, and then put the die on a piece of paper (see Figure 1 for an overview of the setup). They then did the same with the remaining four dice. They were then asked to count the number of stars they rolled with the five dice, to put all dice back into the cup, and to report the number of stars to the experimenter. Before rolling the dice, the experimenter reminded children to be truthful by saying, “Remember you have to truthfully report what you have rolled.” In the collaboration condition, the partner was then invited back into the room, the experimenter announced the first child's outcome, and distributed the rewards accordingly. In the solo condition, the experimenter handed out the rewards before the partner was invited back into the room. Children in the solo condition were thus unaware of how many stickers the other child received to eliminate competitive motives and the well‐documented aversion to disadvantageous inequities in children of the studied age range (Blake et al. 2015). Subsequently, the second child played the game in the same way while the first child waited outside. This procedure was repeated five times to allow for the analysis of round effects, which we expected might differ between conditions.

In both conditions, children were asked not to talk to each other when changing roles to prevent them from influencing each other. Moreover, before each round, children were reminded to report the outcome truthfully.

We used different stickers in each round to keep motivation high. If children reported fewer than five stars over the course of the experiment, the experimenter gave them a total of five stickers at the end. Otherwise, they were rewarded according to the reported outcomes.

Children's responses for each round were coded in real‐time by the experimenter.

### 2.3 Posttest Survey

We assessed the cultural orientation of parents using a survey conducted after the main study. For this purpose, we administered a shortened version of the Cultural Orientation Survey (Singelis et al. 1995; Triandis and Gelfand 1998) to parents from the classes in which the study was conducted. Note that the questionnaire was entirely anonymous, and we could not link parental responses to the game behavior of individual children. Instead, the purpose of including the questionnaire was to allow for a more detailed description of the cultural context and to test if parents of children in Kazakh‐speaking classes differed in their cultural orientation from parents in Russian‐speaking classes. The survey contained questions addressing horizontal and vertical individualism, as well as horizontal and vertical collectivism. Vertical orientation involves acceptance of inequality, while horizontal orientation emphasizes equality. The questions were translated into Kazakh and Russian by the first author and back‐translated into English by independent native speakers. The final sample included 76 parents (40 from Kazakh‐speaking classes).

### 2.4 Statistical Analyses

The dependent variable was the number of stars children reported to have rolled in a given round. The chance level to obtain a star was 1/6 per die, with an average of 0.83 stars expected per round (1/6 × 5 = 0.83). While we cannot know if individual children cheated (children played unobserved), any significant deviations from chance and differences between conditions, language groups, or rounds can be attributed to different cheating rates (Abeler et al. 2019; Fischbacher and Föllmi‐Heusi 2013; Shalvi et al. 2025).

First, we used one‐sample *t*‐tests to compare children's reporting rates to chance (averaged on the dyad level to account for data nonindependence). A preregistered preliminary analysis then investigated the effects of gender, age, and school on children's tendency to report stars. These factors were not included in the main analysis, as they did not significantly affect the dependent variable (all *p* > 0.100). For our main analysis, we employed linear mixed models (LMM) to analyze the effect of condition, language group, round, and their interactions on the number of stars children reported. We included the random effects of participant ID and dyad and the random slopes of the round nested within participant ID and dyad (for an alternative approach treating each die roll as a binomial outcome, see the online supplementary information [SI]).

Analyses were fitted in R (R Core Team 2023) using the function “lmer” of the R‐package lme4 (Bates et al. 2014). We first compared the full model just described to a null model not containing the predictors condition, language group, and round but retaining all random effects and random slopes components. We conducted hypotheses‐driven tests of individual predictors using the function “drop1” only after this full‐null model comparison revealed a significant effect of the predictors of interest combined (Forstmeier and Schielzeth 2011).

To investigate whether children's responses in the collaboration condition were affected by their partner's previous outcomes, we fitted additional LMMs, including the outcome the partner reported on the previous round or the average outcome the partner reported on all previous rounds as an additional predictor. We further included the interaction between language and the partner's reported outcome to examine whether partner influence differed between Russian‐speaking and Kazakh‐speaking children. We excluded the first round of the first child from these analyses since no partner influence could have occurred.

To compare parents’ individualism and collectivism scores in the posttest survey, we used two‐sample *t*‐tests. For further details on all analyses, including model coefficients, see Tables S3, S4, and S7 in the SI.

The study was preregistered at as predicted (https://aspredicted.org/739_STL) and the data can be accessed at https://osf.io/zhyxs/?view_only=50f537c9d3344777bd247d5fb8b8296e.

## 3 Results

**FIGURE 2:**
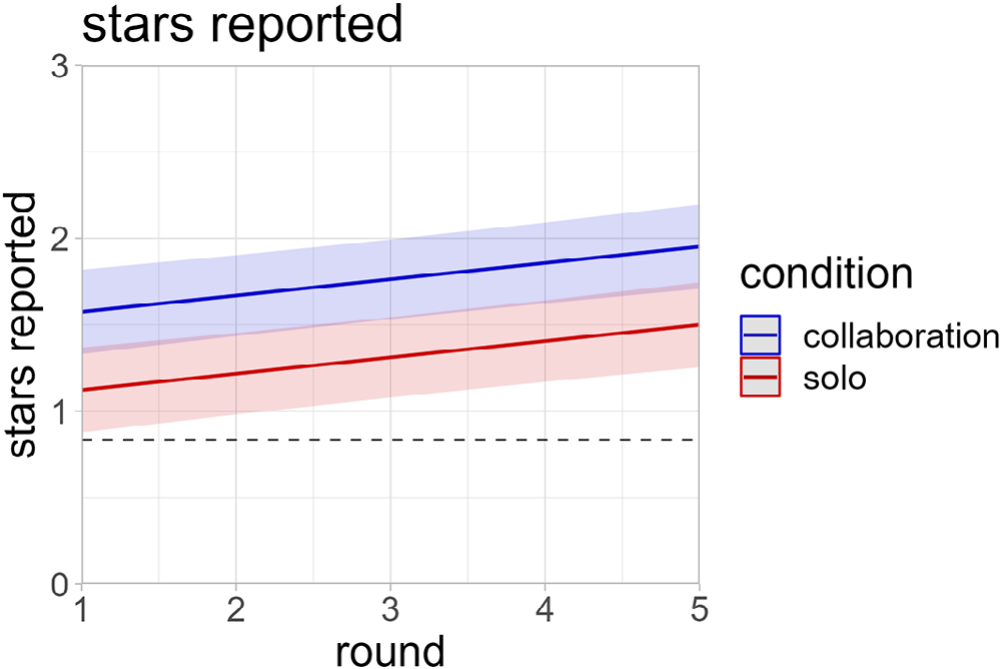
Fitted values of stars reported in the collaboration condition and the solo condition over the five rounds of the experiment. Shaded areas represent 95% confidence intervals. The dashed line represents the chance level had children reported truthfully.

**FIGURE 3:**
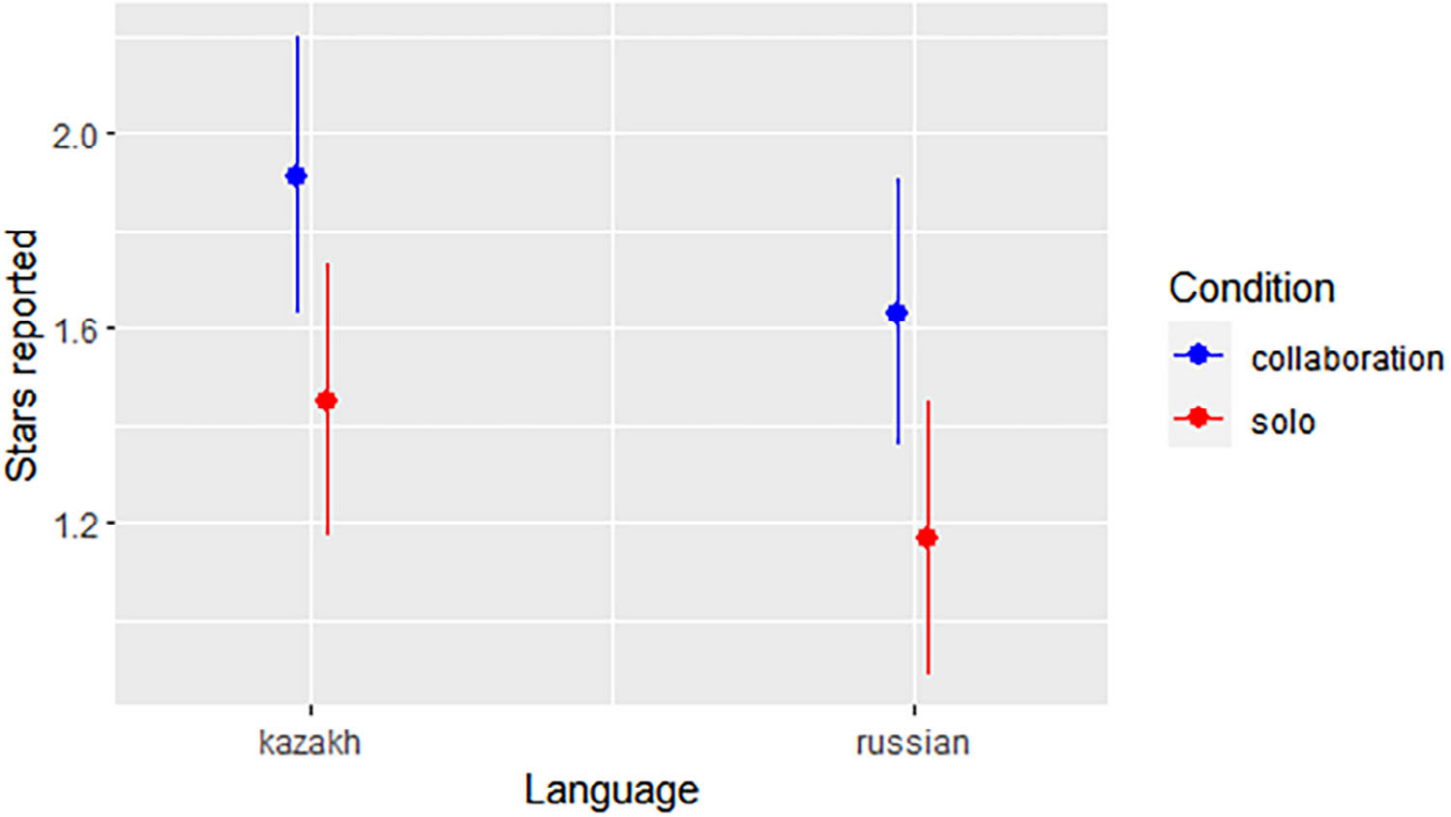
Fitted values of stars reported in two language groups and conditions based on linear mixed‐effects models. Vertical lines represent 95% confidence intervals.

On average, children reported 1.54 stars per round, which significantly exceeds the chance level of 0.83, *t*(97) = 7.96, *p* < 0.001. Over‐reporting occurred in both conditions and in both language groups (*p* < 0.001 for all four *t*‐tests). The full model, containing the three main predictors—condition, language group, and round—fit the data significantly better than the null model, *χ* ^2^(7) = 30.66, *p* < 0.001. Follow‐up analyses revealed no significant three‐way or two‐way interaction effects between the main predictors (*p* > 0.130). However, there were three main effects. First, children reported significantly more stars (i.e., were more likely to cheat) in the collaboration condition compared to the solo condition (*χ* ^2^(1) = 6.53, *p* = 0.011, Figure 2). That is, children reported more stars when they and their partners benefited together than when they benefited individually. Second, children in the Russian‐speaking group reported significantly fewer stars than children in the Kazakh‐speaking group (*χ* ^2^(1) = 5.74, *p* = 0.017, Figure 3). Lastly, children reported more stars with increasing round number (*χ* ^2^(1) = 13.26, *p* < 0.001, Figure 2). An alternative analysis coding the dependent variable as a binomial outcome by assigning a 1 for each die a participant claimed to have rolled a star and a 0 for all others produced virtually identical results (see SI for details).

Analyses investigating whether children in the collaboration condition influenced each other revealed that, in a given round, the number of stars children reported was not significantly impacted by the number of stars their partner reported in the previous round (*χ* ^2^ (2) = 3.53, *p* = 0.170), or by the average number of stars reported by the partner across all previous rounds (*χ* ^2^(2) = 1.34, *p* = 0.510). Furthermore, whether children played first or second did not have a significant effect (*χ* ^2^(1) = 0.27, *p* = 0.599), and children showed a tendency to report more stars in the collaboration condition than in the solo condition already in round 1 (*χ* ^2^(1) = 3.15, *p* = 0.076). Similarly, children from Kazakh‐speaking classes reported more stars than children from Russian‐speaking classes already in round 1 (*χ* ^2^(1) = 5.33, *p* = 0.021).

An exploratory analysis revealed no significant main effect of age or interactions between age and language group or round (*p* > 0.3). There was a marginally nonsignificant interaction between age and condition, *χ* ^2^(1) = 3.80, *p* = 0.051: Age had no significant effect in the solo condition, *χ* ^2^(1) = 0.09, *p* = 0.761, but a marginal negative effect in the collaboration condition, *χ* ^2^(1) = 3.40, *p* = 0.065, indicating that the condition effect in our original analysis was more strongly driven by younger children. However, since these analyses were not preregistered and did not reach significance, they should be interpreted with caution (see SI for details).

### 3.1 Posttest Survey Results

Parents of children attending Kazakh‐speaking classes scored higher on collectivism (*M* = 8.03, SD = 1.38) than parents of children attending Russian‐speaking classes (*M* = 7.09, SD = 1.41), *t*(74) = 2.94, *p* = 0.004. Moreover, parents of children attending Kazakh‐speaking classes also scored significantly higher on individualism (*M* = 7.73, SD = 1.47) than parents of children attending Russian‐speaking classes (*M* = 6.41, SD = 1.58), *t*(74) = 3.80, *p* < 0.001.

## 4 Discussion

Both Kazakh‐ and Russian‐speaking children were more likely to cheat when they and their partner benefited together compared to when they benefited alone, supporting our first hypothesis that collaborative goals can compromise honesty early in development. These findings are consistent with the conjecture that the commitments emanating from collaborative interactions can undermine adherence to norms and suggest that even young children might treat collaboration as a moral currency that can be traded for honesty (Weisel and Shalvi 2022). They also complement prior research on collaborative dishonesty (Leib et al. 2021; Weisel and Shalvi 2015) by demonstrating this phenomenon among a rarely tested population characterized by both collectivistic and individualistic orientations.

Interestingly, there was no evidence that the outcomes the partner reported or whether children played first or second affected children's tendency to cheat in the collaboration condition, suggesting that the condition effect cannot be explained by children copying or otherwise influencing each other.

The overall stakes were identical in the individual and the collaborative context: across conditions, children could generate two stickers per die roll. However, for individual children the personal incentives to cheat were higher in the solo condition (2 stickers) than in the collaboration condition (1 sticker). The fact that children still showed a higher tendency to overreport in the collaboration condition despite lower individual incentivization speaks to the powerful impact that collaborative goals have on children's decision‐making. One alternative explanation is that children in the solo condition (more so than children in the collaboration condition) may have derived diminishing marginal utility from cheating over time, such that the amount of satisfaction produced by winning additional stickers declined with the number of stickers already gained. However, across conditions, there were almost identical increases in reporting stars over rounds, suggesting that, to the contrary, children actually became more motivated to cheat as the experiment progressed.

The findings thus support the idea that children sometimes prioritize benefiting together with a partner over behaving honestly. Generating mutual benefits might also have provided children with a (moral) justification for acting dishonestly while maintaining a positive self‐image as a good collaborator (Mazar et al. 2008; Weisel and Shalvi 2022). Rather than being prosocially motivated, the condition effect might thus also be underpinned by self‐serving motivations. Disentangling the relative contributions of these factors is an important topic for future research.

The second main finding was the effect of language group. Similar to children from Western and East and South Asian populations (Heyman et al. 2015; Kanngiesser et al. 2024; Liu et al. 2022; Zhao et al. 2021), over‐reporting occurred frequently in both language groups. Yet, children in Kazakh‐speaking classes were more likely to overreport stars than children in Russian‐speaking classes. The different rates of over‐reporting cannot be linked to any particular ethnicity—Russian‐speaking classes were highly heterogeneous, with a majority of ethnic Kazakh children. And while dyad heterogeneity could have plausibly affected over‐reporting in the collaboration condition, the language group effect was consistent across conditions.

One explanation could be that children in Kazakh‐ and Russian‐speaking classes may come from environments emphasizing different social‐cultural values. Indeed, parents of children attending Kazakh‐speaking classes scored higher on collectivism than parents of children attending Russian‐speaking classes, corresponding to the finding that dishonesty in adults was correlated with country‐wide measures of collectivism orientations (Gächter and Schulz 2016; Cohn et al. 2019). Yet, these results are difficult to interpret given that parents of children attending Kazakh‐speaking classes also scored higher on individualism. While documenting differences in cultural orientation between parents in the two language groups, it remains unclear how they may have contributed to children's cheating.

Recent research on value priorities in Kazakhstani young adults reported that Russian‐speakers valued independence more than Kazakh‐speakers. Crucially, Russian‐speakers also showed a greater tendency to value honesty (Zharkynbekova et al. 2025). These results correspond to the current finding that Russian‐speaking children were less likely to cheat overall and raise the interesting possibility that differences in value priorities are already manifest and affect cheating rates in young children.

Another possibility is that different linguistic environments affected children's behavior. In adults, honesty was found to be higher in speakers of languages that prohibit dropping the first‐person pronoun “I” (Cohn et al. 2019)—a linguistic feature associated with emphasizing the individual and stressing personal responsibility (E. S. Kashima and Y. Kashima 1998). Interestingly, while we are not aware of research specifically examining first‐person pronoun use in Kazakh, first‐person pronouns are not commonly used in Turkish, a closely related Turkic language (Uygun 2022), whereas, in Russian, dropping personal pronouns is partially permitted (Bizzarri 2015). Moreover, research with English‐speaking children has shown that a collaborative “we”‐framing (compared to an individualistic “you”‐framing) can affect young children's social decision‐making (Vasil and Tomasello 2022), suggesting that, even though the instructions were carefully matched, subtle linguistic cues may have resulted in different game interpretations.

The current study echoes arguments for studying variation at multiple levels. Many studies compare populations only on a country level, and, while useful, this approach risks missing important within‐country variation (Amir and McAuliffe 2020; Lamba and Mace 2011). By documenting variation between populations identical on many variables commonly considered as potential influences on dishonesty (e.g., GDP, corruption indices, political organization, school environment), the current study allows for more narrow interpretations and more targeted future hypotheses.

While collaborative dishonesty has been repeatedly reported in North American and European adults, an interesting question is whether this phenomenon might develop earlier (or be more common) in regions characterized by a high prevalence of rule violations and strong social interdependencies. Addressing this issue would be a fruitful avenue for future research. Future studies could also employ simplified designs suitable for younger children to explore the first origins of collaborative dishonesty and include questionnaires assessing children's normative reasoning about conflicts between honesty norms and cooperative goals. Further manipulations of dyad composition, cooperative incentives, and costs of cheating could help ascertain the degree to which the current findings generalize across contexts and reward structures.

In summary, the current study demonstrates that Kazakhstani children are more likely to cheat in collaborative than in solo settings, indicating that the motive to generate benefits with others collaboratively—a central aspect of human sociality—can be implicated in the erosion of norms. The fact that collaborative dishonesty is already present in young children speaks to the potential of collaborative motives in compromising honesty. Finally, the differences between populations underscore that social‐cultural factors can influence cheating tendencies from early in development (Kanngiesser et al. 2024), thus highlighting the importance of a developmental approach for understanding variation in cheating levels.

## Author Contributions

**Akzira Abuova**: conceptualization, investigation, writing – original draft, methodology, visualization, formal analysis, project administration, Data curation. **Laura Tietz**: conceptualization, methodology, writing – review and editing. **Sebastian Grueneisen**: conceptualization, methodology, formal analysis, resources, writing – original draft, writing – review and editing, supervision.

## Ethics Statement

The study was approved by the Ethics Advisory Board of Leipzig University.

## Conflicts of Interest

The authors declare no conflicts of interest.

## Supporting information




**Supporting File 1**: desc70080‐sup‐0001‐SuppMat.docx

## Data Availability

All primary data and analysis scripts are publicly available at https://osf.io/zhyxs/?view_only=50f537c9d3344777bd247d5fb8b8296e.
