# Supplementary material for: Collaborative Dishonesty: Children Are More Likely to Cheat When They Benefit Together
Source: Dev Sci. 2025 Oct 13;28(6):e70080. doi: 10.1111/desc.70080 (PMC12519045; doi:10.1111/desc.70080)
Supplement: Supplementary file 1 — Supporting File 1: desc70080‐sup‐0001‐SuppMat.docx [file DESC-28-e70080-s001.docx]

**Supplementary Information for**:

Collaborative Dishonesty: Children are More Likely to Cheat When They Benefit Together

Table of Contents

1. Information on the additional analysis....................................................................................2

2. Supplementary Tables.............................................................................................................5

**1.** **Information on the additional analysis**

**1.1 Analysis investigating age effects**

We conducted an additional exploratory analysis investigating potential age effects. To this end, we extended our previous main-effects model by adding the main effect of age (in months) as well as its two-way interactions with condition, language group, and round to our main effects model, resulting in the following model:

model =lmer(number.of.stars ~ language*age.in.months + condition*age.in.months + round*age.in.months + (1|ID.number) + (1|dyad.number) + (0+round|ID.number) + (0+round |dyad.number))

This analysis revealed no significant main effect of age, χ2(1) = 1.05, p = .304, and no significant interactions between age and language group, χ2(1) = 0.01, p = .934, or age and round, χ2(1) = 0.31, p = .577. There was marginally non-significant interaction between age and condition, χ2(1) = 3.80, p = .051 (see table S2). Follow-up analyses revealed that age had no significant effect in the solo condition, χ2(1) = 0.09, p = .761, and a marginal negative effect in the collaboration condition, χ2(1) = 3.40, p = .065, indicating that the condition effect in our original analysis was more strongly driven by younger children in the sample. However, these analyses were not preregistered, no corrections for multiple testing were applied (e.g., no full-null model comparisons), and none of the effects reached significance. These results should thus be interpreted with caution.

**1.2 Analysis treating the dependent variable as a binomial outcome**

We conducted an additional analysis in which the dependent variable was coded as a binomial outcome. For each round, participants were assigned 5 binary data points corresponding to the number of stars they reported in that round (i.e., a value of 1 for each star they reported, and 0 for all others).

We then fitted a Generalized Linear Mixed Model (GLMM) with binomial error structure, which, as in our original model, included the predictors condition, language group, and round as well as their interactions. The model also included the random intercepts of participant ID and dyad as well as the random slopes components of round nested within participant ID and dyad. The model specification was as follows:

model=glmer(star ~ condition*language*round + (1|ID.number) + (1|dyad.number) + (0+round|ID.number) + (0+round|dyad.number), family = binomial)

As with our original analysis, we first conducted a full–null model comparison, followed by single-term deletions. The results of this analysis are consistent with our original findings. Specifically, the full model provided a better fit than the null model, χ2(7) = 23.23, p = .002. Follow-up analyses indicated no significant three-way or two-way interactions (all p > .1).

However, all three main effects remained significant: Children were more likely to report stars in the collaboration condition than in the solo condition, χ2(1) = 6.61, p = .010, Kazakh-speaking children were more likely to over-report stars than Russian-speaking children, χ2(1) = 5.14, p = .023, and children were more likely to report stars with increasing round number, χ2(1) = 6.11, p = .013 (see table S3.1).

**2. Supplementary Tables**

Table S1. Preliminary Analysis

| Predictor | Estimate | St. Error | 95% CI | 𝜒^2^ | DF | *p* |
| --- | --- | --- | --- | --- | --- | --- |
| Age (in months) | -0.014 | 0.011 | -0.035, 0.007 | 1.76 | 1 | .185 |
| Gender(male) | -0.185 | 0.179 | -0.531, 0.162 | 1.12 | 1 | .290 |
| School (B) | 0.121 | 0.176 | -0.220, 0.462 | 0.49 | 1 | .486 |

Reference categories in brackets

Table S2. Age Effects

| Predictor | Estimate | St. Error | 95% CI | 𝜒^2^ | DF | *p* |
| --- | --- | --- | --- | --- | --- | --- |
| Full-null comparison |  |  |  | 5.32 | 4 | .256 |
| Age*language | 0.001 | 0.019 | -0.036, 0.039 | 0.01 | 1 | .934 |
| Age*condition | 0.037 | 0.019 | -0.000, 0.073 | 3.80 | 1 | .051 |
| Age*round | 0.002 | 0.003 | -0.008, 0.004 | 0.31 | 1 | .577 |
| Age (in months) | -0.027 | 0.019 | -0.064, 0.010 | 1.05 | 1 | .304 |

Table S3. Main Analysis 1 – Condition, Language, Effect (LMM)

| Predictor | Estimate | St. Error | 95% CI | 𝜒^2^ | DF | *p* |
| --- | --- | --- | --- | --- | --- | --- |
| Full-null comparison |  |  |  | 26.73 | 7 | .000 |
| Cndition*language* round | 0.067 | 0.102 | -0.129, 0.264 | 0.46 | 1 | .497 |
| Condition*language | 0.452 | 0.452 | -0.135, 1.044 | 2.29 | 1 | .130 |
| Condition*round | -0.054 | 0.050 | -0.153, -0.044 | 1.19 | 1 | .276 |
| Language*round | 0.068 | 0.50 | -0.030, 0.166 | 1.85 | 1 | .174 |
| Language (Russian) | -0.369 | 0.153 | -0.668, -0.069 | 5.74 | 1 | .017 |
| Condition (solo) | -0.393 | 0.170 | -0.692, -0.094 | 6.53 | 1 | .011 |
| Round | 0.094 | 0.025 | 0.044, 0.144 | 13.26 | 1 | .000 |

Reference categories in brackets

Table S3.1. Additional Analysis – Condition, Language, Round Effect (GLMM with binomial error structure)

| Predictor | Estimate | St. Error | 95% CI | 𝜒^2^ | DF | *p* |
| --- | --- | --- | --- | --- | --- | --- |
| Full-null comparison |  |  |  | 23.23 | 7 | .002 |
| Cndition*language* round |  |  |  | 0.60 | 1 | .438 |
| Condition*language | 0.506 | 0.312 | -0.173, 1.127 | 2.60 | 1 | .107 |
| Condition*round | -0.065 | 0.060 | -0.185, 0.041 | 1.17 | 1 | .276 |
| Language*round | 0.075 | 0.060 | -0.045, 0.194 | 1.24 | 1 | .213 |
| Language (Russian) | -0.367 | 0.158 | -0.679, -0.052 | 5.14 | 1 | .023 |
| Condition (solo) | -0.414 | 0.158 | -0.726, -0.102 | 6.61 | 1 | .010 |
| Round | 0.080 | 0.031 | 0.017, 0.140 | 6.11 | 1 | .013 |

Reference categories in brackets

Table S4. Main Analysis 2 – Partner’s Behavior in the Previous Round in the Collaboration Condition

| Predictor | Estimate | St. Error | 95% CI | 𝜒^2^ | DF | *p* |
| --- | --- | --- | --- | --- | --- | --- |
| Full-null comparison |  |  |  | 3.54 | 2 | .170 |
| Partner’s previous outcome*language | 0.151 | 0.097 | -0.040, 0.340 | 2.39 | 1 | .122 |
| Partner’s previous outcome | 0.058 | 0.048 | -0.054, 0.153 | 1.14 | 1 | .285 |
| Language (Russian) | -0.510 | 0.252 | -1.006, 0.017 | 4.11 | 1 | .043 |

Reference categories in brackets

Notes: We tested if the outcome the partner reported on the previous round affected reporting of stars in the collaboration condition. We added the variable “partner’s previous outcome” which indicates what the partner reported in the round before. We did not include the first round of child 1 (i.e., the child who played first), as no partner influence could have occurred.

Table S5. Main Analysis 2 – Partner’s Behavior in all Previous Rounds in the Collaboration Condition

| Predictor | Estimate | St. Error | 95% CI | 𝜒^2^ | DF | *p* |
| --- | --- | --- | --- | --- | --- | --- |
| Full-null comparison |  |  |  | 1.34 | 2 | .510 |
| Partner's previous average* language | -0.008 | 0.170 | -0.342, 0.324 | 0.004 | 1 | .952 |
| Partner’s previous average | 0.108 | 0.085 | 0.113, 0.274 | 1.34 | 1 | .246 |
| Language (Russian) | -0.482 | 0.254 | -0.979, 0.015 | 3.62 | 1 | .057 |

Reference categories in brackets

Notes: We tested if the average number of stars reported by the partner over all previous rounds affected the outcome children reported in the collaboration condition. We created the variable “partner's previous average” indicating the mean number of stars reported by the partner on all previous rounds.

Table S6. Exploratory Analysis - Round 1 Behavior

| Predictor | Estimate | St. Error | 95% CI | 𝜒^2^ | DF | *p* |
| --- | --- | --- | --- | --- | --- | --- |
| Full-null comparison |  |  | -0.582, 0.029 | 8.54 | 3 | .036 |
| Language (Russian) | -0.369 | 0.157 | -0.669, -0.057 | 5.34 | 1 | .021 |
| Condition (solo) | -0.277 | 0.157 | -0.692, -0.094 | 3.15 | 1 | .076 |
| Child role (C2) | -0.079 | 0.150 | -0.372, 0.218 | 0.28 | 1 | .599 |

Reference categories in brackets

Notes: We tested whether the effect of condition and language was already present on round 1.

Table S7. Post-test survey – Cultural Orientation in Kazakh-speaking and Russian-speaking Parents

| Variable | t-statistics | DF | 95% CI | *p* |
| --- | --- | --- | --- | --- |
| Individualism | 3.80 | 74 | 0.632, 2.024 | .000 |
| Collectivism | 2.94 | 74 | 0.304, 1.585 | .004 |

Notes: A two-sample t-test was conducted to compare the cultural orientation scores between Kazakh-speaking and Russian-speaking parents. Results showed that parents of children in Kazakh-speaking classes scored significantly higher on both collectivism and individualism compared to parents of children in Russian-speaking classes.
